# Variations in Eyeball Diameters of the Healthy Adults

**DOI:** 10.1155/2014/503645

**Published:** 2014-11-05

**Authors:** Inessa Bekerman, Paul Gottlieb, Michael Vaiman

**Affiliations:** ^1^Department of Roentgenology, Assaf Harofeh Medical Center, Affiliated to Sackler Faculty of Medicine, Tel Aviv University, 70300 Zerifin, Israel; ^2^Department of Otolaryngology-Head and Neck Surgery, Assaf Harofeh Medical Center, Affiliated to Sackler Faculty of Medicine, Tel Aviv University, 33 Shapiro Street, 70300 Zerifin, Israel

## Abstract

The *purpose* of the current research was to reevaluate the normative data on the eyeball diameters. *Methods*. In a prospective cohort study, the CT data of consecutive 250 adults with healthy eyes were collected and analyzed, and sagittal, transverse, and axial diameters of both eyeballs were measured. The data obtained from the left eye and from the right eye were compared. The correlation analysis was performed with the following variables: orbit size, gender, age, and ethnic background. *Results*. We did not find statistically significant differences correlated with gender of the patients and their age. The right eyeball was slightly smaller than the left one but this difference was statistically insignificant (*P* = 0.17). We did not find statistically significant differences of the eyeball sizes among the ethnicities we dealt with. Strong correlation was found between the transverse diameter and the width of the orbit (*r* = 0.88). *Conclusion*. The size of a human adult eye is approximately 24.2 mm (transverse) × 23.7 mm (sagittal) × 22.0–24.8 mm (axial) with no significant difference between sexes and age groups. In the transverse diameter, the eyeball size may vary from 21 mm to 27 mm. These data might be useful in ophthalmological, oculoplastic, and neurological practice.

## 1. Introduction

For decades, computed tomography (CT) has been routine investigation in ophthalmology and ophthalmoneurology. Currently, CT investigations in ophthalmology are very detailed [1–3]. Thus, gross anatomy of the eye attracts less attention though it is useful not only in cases of eye diseases but in some neurological conditions as well [4].

In ophthalmology, eyeball trauma, cancer, congenital glaucoma, retinal blastoma, and some other disorders can change the size of the eyeball [5]. The oblate/prolate shapes of the eyeball can be traced already in newborns and can influence the development of myopic refractive errors [6]. Microphthalmos is a disorder of the eye, often congenital, due to arrest in growth of the ocular tissues. When the eyeball is visibly small, the diagnosis is simple but in border cases the distinction between the normal size and the pathologically small size of the eyeball requires precise knowledge of the normal anatomy. This distinction is not well defined yet especially for cases of the posterior microphthalmos [7].

In neurology, current interest in optic nerve sheath diameter (ONSD) and its possible connection with the intracranial pressure monitoring requires precise size measurements also. It was shown that the calculation of an index when ONSD is divided by the eyeball transverse diameter presents precise normative database for ONSD intracranial pressure measurement technique [8]. Therefore precise knowledge about normative size of the eyeball is as important as measurement of the normative ONSD. That is why we think it is necessary to refresh our knowledge about the eyeball diameters as they can be measured by a routine CT investigation in a clinic.

The first edition of Henry Gray's “Anatomy Descriptive and Surgical” of 1858 mentioned that “the antero-posterior diameter of the eyeball measures about an inch,* [sic]* exceeds the transverse diameter by about a line” [9]. In 1912, the generally accepted average measurements of the eyeball diameters taken by various authors were 24.26 mm for the anteroposterior diameter, 23.7 mm for the transverse diameter, and 23.57 mm for the vertical diameter [10].

To the beginning of the XX century, it was well established that the size of the eyeball is variable. At that time, however, only age, gender, and refraction were respected as causes for these variations [11]. In 1970, it was already well established that the axial length is different in cases with myopia (24.61 ± 1 mm), emmetropia (23.40 ± 1.38 mm), and hypermetropia (22.53 ± 1.02 mm) [12]. At present, researches describe a more complicated picture indicating that there are considerable individual variations of shape and size in myopic eyes and that there may be different types of myopia [13].

While specific books on the anatomy of the eye dedicated the whole chapter on the subject [14], there is no universal agreement on the normative data. The current state of knowledge at the level of Gray's Anatomy postulates that “the ocular vertical diameter (23.5 mm) is rather less than the transverse and anteroposterior diameters (24 mm)” [15]. This statement was slightly changed in the manual on “Comprehensive Ophthalmology” (2007), which indicated somewhat smaller eyes with the dimensions of an adult eyeball as 24 mm (axial, anteroposterior) × 23.5 mm (horizontal, transverse) × 23 mm (vertical, sagittal) [16]. Some current manuals and general works on ophthalmology and neuroophthalmology do not indicate normative dimensions of the eyeball even when buphthalmos and microphthalmos are described or oculoplastic matters are discussed [17–19].

As for variations, the generally accepted statement at the level of manuals on ophthalmology is that the eyeball diameters “differ among adults by only one or two millimeters” [20].

The purpose of the current research was to check all these statements with the help of current data obtained by computed tomography (CT) technique. In addition to that, we planned to investigate possible correlation between the eyeball size and the size of the orbit because to our knowledge it was not done yet.

## 2. Materials and Methods

In a prospective cohort study, the CT data of consecutive 250 adult patients (18+) that were admitted to the department of roentgenology at our medical center from 2011 to 2012 were collected and analyzed. The study protocol conformed to the ethical guidelines of the 1975–2000 Declaration of Helsinki as reflected* a priori* after approval by the institution's Helsinki committee. The cohort consisted of the cases who were scheduled and underwent the CT investigation that included the head and neck region. In all cases, the CT investigation was requested by the emergency room because of the various medical conditions. The cases that proved not to be connected with ophthalmological or neurological pathology were selected for the current study.

Exclusion procedure was organized in two steps. First, the patients with documented ophthalmologic or neuroophthalmologic disorders were excluded as well as patients with injuries around the eyeballs and the orbits. Second, the selected patients were examined by an ophthalmologist to exclude overlooked eye disorders including squint, exophthalmos, and astigmatism. After that, the selected patients were divided into three refraction groups: (I) patients with myopia (*n* = 56), (II) patients with emmetropia (*n* = 118), and (III) patients with hypermetropia (*n* = 76). Myopia was defined as a spherical equivalent of at least −0.5 D, hyperopia a spherical equivalent of at least +2.0 D, and astigmatism a cylinder of at least −1.0 D in at least one eye. In Group (I), some patients had only one myopic eye while the other eye was emmetropic. Therefore, the distribution of the eyes within these groups was as follows: (I) myopic eyes *n* = 109; (II) emmetropic eyes *n* = 239; and (III) hypermetropic eyes *n* = 152. The patient flow was as follows: from the 362 consecutive patients, 74 were excluded at the first step and 38 were excluded at the second step. The data collection was stopped when we obtained 250 cases with healthy eyes.

**Figure 1:**
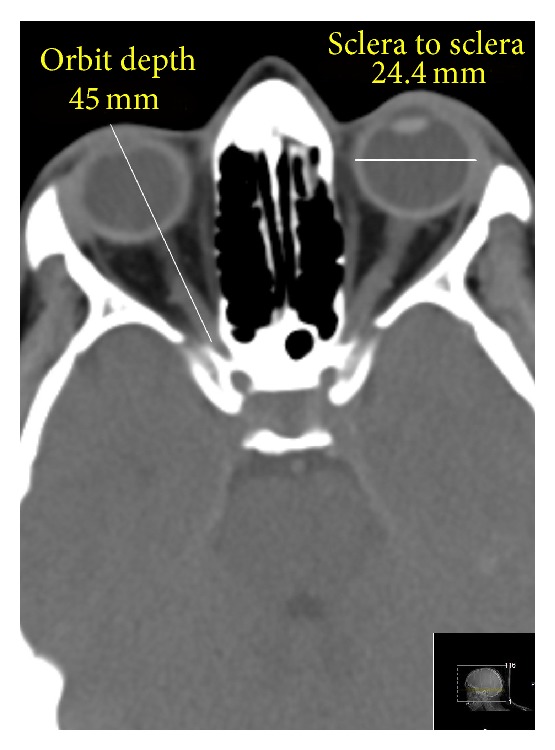
The retina to retina transverse diameter of the eye measured by computed tomography.

**Figure 2:**
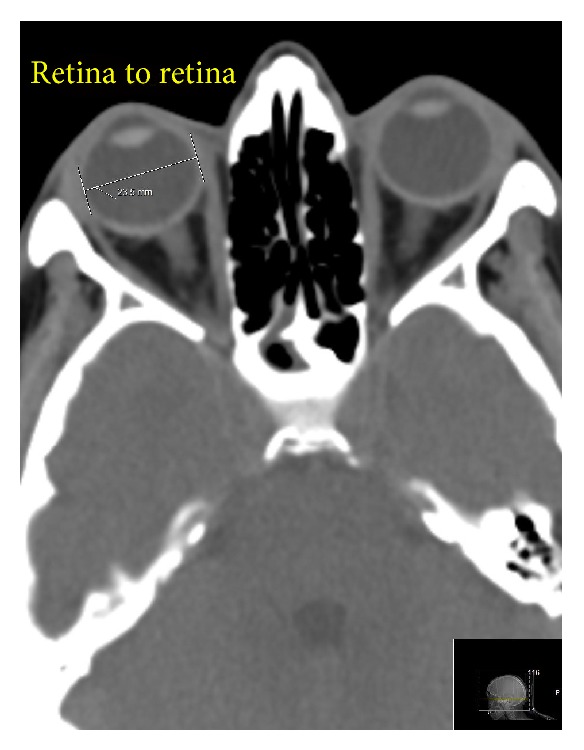
The cornea to optic canal anterior opening depth of the orbit and the sclera to sclera diameter of the eyeball.

All the CT scans were obtained by the Philips Brilliance iCT 256-Slice Helical Scanner (Philips, The Netherlands) with NanoPanel 3D spherical detectors. The standard Philips protocols for head and neck imaging were implemented in all cases, single slice section 3 mm. When the CT scans were obtained, sagittal, transverse, and axial (anterior-to-posterior) diameters of both eyeballs were measured by the Philips computer program (spine window, middle third; window parameters: WW 60, WL 360, accuracy: 1 pixel). All measurements were made using the same window, contrast, and brightness. The sagittal and transverse diameters were measured twice, by the outer edge of the fibrous coat (sclera to sclera) and by the inner edge of the fibrous coat (retina to retina) (Figure 1), and the axial diameter was measured from cornea to sclera. The height and width of orbital margin were measured by superficial bony margins but the depth of the orbit was measured from cornea to the anterior opening of the optic canal for correlation purposes (Figure 2).

The error margin was expressed by the technical error of measurement (TEM) to calculate the intraevaluator variability and interevaluator variability between two evaluators. The same equipment and methodological procedures for measurements were adopted by both evaluators.

### 2.1. Analysis

A within-group repeated measures experimental statistical analysis was used to test the variables. To verify the normality of the data, normal probability plots and basic descriptive statistics (mean, standard deviation (SD), min, and max) were calculated for every variable (three eyeball diameters, three orbit measurements). The data obtained from the left eye and from the right eye were compared. The correlation analysis was performed with the following variables: orbit size, gender, age group (group (I): 18–30; group (II): 30–65; group (III): 65+), and ethnic background. The data were statistically evaluated by three-dimensional analysis of variance, SPSS, Standard version 17.0 (SPSS, Chicago, IL, 2007), and correlations were evaluated with *χ* ^2^ criterion using 95% confidence interval. The level of significance for all analyses was set at *P* < 0.05.

## 3. Results

In our cohort, there were 134 females and 116 males and age range was from 18 to 93 (mean 47). Altogether, 500 eyeballs were measured. For the TEM calculation, two measurements were obtained from each eye (*n* = 1000 measurements). The difference between the first and second measurements was then determined and the relative TEM (technical error of measurement expressed in %) was calculated to be 2.56 (acceptable) for intraevaluator TEM and 3.47 (acceptable) for interevaluator TEM.

The ethnic background of the patients was as follows:

- Jews and half-Jews of European origin (Ashkenazi): 56,
- Jews and half-Jews of Middle or Near Eastern and Central Asian origin: 52,
- Jews and half-Jews of Northern African origin (Sephardi): 47,
- various European and North American nationalities: 56,
- Palestinian Arabs: 22,
- Ethiopians and other African nationalities: 17.

Table 1 presents the results of the measurements, and Table 2 presents the comparison and correlation results. We did not find statistically significant differences correlated with gender of the patients (*P* = 0.14) and their age ((I) versus (II), *P* = 0.23; (I) versus (III), *P* = 0.09; (II) versus (III), *P* = 0.33). In our cases, the right eyeball appears to be slightly smaller than the left one in all diameters but these differences were also statistically insignificant (*P* = 0.17 transverse, *P* = 0.23 sagittal, and *P* = 0.44 axial). Finally, we did not find statistically significant differences of the eyeball sizes among the participants of different ethnic backgrounds we dealt with in pairwise comparisons (e.g., (a) versus (b), *P* = 0.42 and (a) versus (d), *P* = 0.25).

The strong correlation existed between the eyeball transverse diameter and the width of the orbit while other diameters did not correlate with the orbit height or depth.

## 4. Discussion

In general, our data show somewhat smaller size of the eyeball that did not reach 24.5 mm either in sagittal or in transverse diameter. Currently, quantitative data are very precise and each 0.1 mm counts. For example, in performing an A-scan practitioners are warned that “corneal compression in contact A-scan reduces the measured axial length by 0.1–0.3 mm, even for a careful user” and that “all the averaged scans should be within 0.2 mm of each other” [17, 21]. When optic nerve sheath diameter is measured for intracranial pressure monitoring, the measurements are also very precise [22]. There is a strong correlation between the eyeball transverse diameter (ETD) and ONSD that can be presented as ONSD/ETD index [8]. In healthy subjects, the ONSD/ETD index equals 0.19 while larger number indicates elevation of the intracranial pressure. The precise knowledge of the normative data on the eyeball dimensions is paramount for such calculations.

The irregularities of the eyeball shape were detected in low myopia and well documented [23, 24]. In general, our findings in quantitative differences between myopic, emmetropic, and hypermetropic eyes support the previously reported data [12, 13, 23, 24].

Discussing the implemented technique of the investigation, CT is widely used in ophthalmology and very often it is an initial investigation in emergency departments. Normal* in vivo* eye dimensions were measured by computed tomography at least since early 1980s. At that time, the reasoning was expressed where CT measurements of the eyeball diameters might underestimate the actual* in vivo* dimensions of the eye [25]. Thirty years after that, a modern CT scan with 1-pixel accuracy measures eyes precisely. Estimating the CT hardware and software that was in our disposal, and taking the technical error of measurement data into account, we suggest that the obtained data are accurate. CT measurements of the eyeball diameters provided by other authors show accurate results also [26]. While CT measurement of the sagittal diameter is somewhat complicated, in cases of transverse (Figure 1) and axial (anteroposterior) diameters we see no obstacles that possibly might affect the accuracy of the measurements. The eyeball, however, is imbedded in the large quantity of fat and delicate connective tissue that in some cases can make scleral surface somewhat unclear when the transverse and the sagittal (vertical) diameters are measured. If any doubts exist, we suggest making two measurements: retina to retina and sclera to sclera.

We agree with the authors stating that the shapes and sizes of the eyeballs varied considerably between subjects [13, 23, 24]. The difference between the eyes of 21 mm and of 27 mm in the transverse diameter is actually more than a half of the centimeter. While the extremes are rare, they still exist. Myopia and hypermetropia change axial diameter of the eye but do not change other diameters [12]. Therefore, for practical measurement of the eyeball size in ophthalmologic or neurologic clinic, we suggest estimating the transverse diameter. The transverse diameter well correlates with the width of the orbit. This diameter therefore can be useful in oculoplastic calculations as well. At the same time, myopic and hypermetropic changes of anteroposterior (axial) diameter have no correlation with the depth of the orbit.

We see the limitation of this research in view of possible differences in eyeball dimensions between patients with different ethnic backgrounds. While in our series we did not find any significant differences in these dimensions among patients of various nationalities that were hospitalized at our clinic, we cannot suggest generalization in this matter. The recent Chinese research suggests, for example, that Asian eyes had smaller anterior segments compared to Caucasian eyes [27]. Another recent article suggests that differences in ocular shape might play a role in the greater propensity for East Asians to develop and progress in myopia compared with Caucasian ethnicities [25]. Additional research might clarify the picture.

## 5. Conclusion

The size of an emmetropic human adult eye is approximately 24.2 mm (transverse, horizontal) × 23.7 mm (sagittal, vertical) × 22.0–24.8 mm (axial, anteroposterior) with no significant difference between sexes and age groups. In the transverse diameter, the eyeball size may vary from 21 mm to 27 mm. Myopia and hypermetropia change the axial diameter significantly that can vary from 20 to 26 mm. The horizontal diameter corresponds with the width of the orbit. These data might be useful in ophthalmological, oculoplastic, and neurological practice.

## Figures and Tables

**Table 1:** Eyeball and orbit diameters measured by computed tomography.

Diameter (mm)	Right eyeball/orbit			Left eyeball/orbit		
	Median ± SD	Max	Min	Median ± SD	Max	Min
Eyeball						
Transverse (r-r)^*^	22.822 ± 1.7	25.5	20.0	22.936 ± 1.8	25.8	19.4
Transverse (s-s)^**^	24.156 ± 1.9	26.8	21.5	24.324 ± 1.9	27.1	20.9
Sagittal (r-r)	22.547 ± 1.2	25.1	20.0	22.604 ± 1.1	24.9	19.7
Sagittal (s-s)	23.799 ± 1.6	26.4	21.2	23.752 ± 1.7	25.6	20.5
Axial, Group I^***^	24.477 ± 1.8	26.2	20.0	24.893 ± 2.2	25.8	20.7
Axial, Group II^****^	23.422 ± 1.9	25.7	20.3	23.562 ± 1.9	25.4	19.9
Axial, Group III^*****^	22.307 ± 2.2	25.7	20.6	22.096 ± 1.9	24.7	20.4
Orbit						
Height of margin	41.075 ± 2.4	44.6	39.4	42.550 ± 2.5	44.8	38.8
Width of margin	35.327 ± 2.2	37.0	32.7	35.862 ± 2.2	37.4	32.9
Depth of orbit	47.732 ± 4.6	55.4	38.8	48.396 ± 4.7	55.7	38.7

^*^(r-r): retina to retina.

^**^(s-s): sclera to sclera.

^***^Myopia.

^****^Emmetropia.

^*****^Hypermetropia.

**Table 2:** Comparison and correlation of the obtained measurements.

Variables compared	*n*	*P* or *r*
Right eyeball versus left eyeball size	250 versus 250	*P* = 0.17
Gender comparison, male versus female	116 versus 134	*P* = 0.14
Pairwise age group comparison:		
Group I versus Group II	86 versus 97	*P* = 0.23
Group I versus Group III	86 versus 67	*P* = 0.09
Group II versus Group III	97 versus 67	*P* = 0.33
Height of margin versus vertical eyeball diameter	500 versus 500	*r* = 0.43
Width of margin versus transverse diameter	500 versus 500	*r* = 0.88
Depth of orbit versus axial, Group I	109 versus 109	*r* = 0.41
Depth of orbit versus axial, Group II	239 versus 239	*r* = 0.32
Depth of orbit versus axial, Group III	152 versus 152	*r* = 0.14
